# RF9 Rescues Cortisol-Induced Repression of Testosterone Levels in Adult Male Macaques

**DOI:** 10.3389/fphys.2021.630796

**Published:** 2021-02-25

**Authors:** Rahim Ullah, Rabia Naz, Aalia Batool, Madiha Wazir, Tanzil Ur Rahman, Ghulam Nabi, Fazal Wahab, Junfen Fu, Muhammad Shahab

**Affiliations:** ^1^Department of Endocrinology, Children’s Hospital, Zhejiang University School of Medicine, National Clinical Research Center for Child Health, Hangzhou, China; ^2^Laboratory of Reproductive Neuroendocrinology, Department of Animal Sciences, Faculty of Biological Sciences, Quaid-i-Azam University, Islamabad, Pakistan; ^3^Key Laboratory of Animal Physiology, Biochemistry and Molecular Biology of Hebei Province, College of Life Science, Hebei Normal University, Shijiazhuang, China; ^4^Department of Biomedical Sciences, Pak-Austria Fachhochschule: Institute of Applied Sciences and Technology, Haripur, Pakistan

**Keywords:** glucocorticoids, testosterone, HPG-axis, RF9, monkey

## Abstract

Cortisol inhibits hypothalamic-pituitary-gonadal (HPG) axis whereas RF9, a potent agonist of kisspeptin receptor (GPR54) activates HPG-axis during fasting-induced stress and under normal physiological conditions. However, the effect of RF9 on the cortisol-induced repressed HPG-axis is not studied yet. This study investigated whether exogenous cortisol-induced repression of the HPG-axis can be rescued by RF9. Six intact adult male rhesus monkeys (*Macaca mulatta*) habituated to chair-restraint were administered hydrocortisone sodium succinate at a rate of 20 mg/kg of body weight (BW) per day for 12 days. Single blood sample was taken by venipuncture from each animal on alternate days for hormones analyses. On experimental day 12, hydrocortisone treated monkeys received a single intravenous bolus of RF9 (*n* = 3) and vehicle (*n* = 3). The animals were bled for a period of 4 h at 60 min intervals from an indwelling cannula in the saphenous vein. RF9 was administered intravenously at the dose of 0.1 mg/kg BW immediately after taking 0 min sample. Plasma cortisol and testosterone concentrations were measured by using specific enzyme immunoassays. Hydrocortisone treatment increased plasma cortisol levels (*P* ≤ 0.0001) and decreased plasma testosterone (*P* ≤ 0.0127) levels. Interestingly, compared to vehicle, RF9 treatment significantly increased plasma testosterone levels at 120 min (*P* ≤ 0.0037), 180 min (*P* ≤ 0.0016), and 240 min (*P* ≤ 0.0001) intervals in the hydrocortisone treated monkeys. From these results, we concluded that RF9 administration relieves the suppressed HPG-axis in term of plasma testosterone levels in the cortisol treated monkeys.

## Introduction

Successful reproduction is the result of a dynamic balance between the stimulatory and inhibitory signals of the central nervous system (CNS) (Wahab et al., 2015; Ullah et al., 2016). In the CNS, a hypothalamic neuropeptide, gonadotropin-releasing hormone (GnRH) is the principal stimulator of the hypothalamic-pituitary-gonadal (HPG) axis in vertebrates (Okubo and Nagahama, 2008). It is a decapeptide, released from a specialized set of neurons located in the preoptic area of the hypothalamus (Okubo and Nagahama, 2008). GnRH then stimulates the secretion of luteinizing hormone (LH) and follicle stimulating hormone (FSH) from the pituitary gland that in turn regulates steroidogenesis and gametogenesis (Ullah et al., 2016).

Gonadotropin-releasing hormone is centrally regulated by various neuropeptides including agouti-related protein (AgRP), neuropeptide Y (NPY), corticotrophin-releasing hormone (CRH), alpha-melanocyte-stimulating hormone (α-MSH), kisspeptin, and gamma-aminobutyric acid (GABA) (Wahab et al., 2015; Ullah et al., 2016). Likewise, peripheral hormones such as leptin, ghrelin, insulin, cortisol, etc., also regulate GnRH neurons (Wahab et al., 2015; Ullah et al., 2016). Kisspeptin stimulates whereas the hypothalamic-pituitary-adrenal axis (HPA-axis) inhibits GnRH neurons (Wahab et al., 2015; Ullah et al., 2016). It is well-known that administration of natural or synthetic glucocorticoids inhibits the secretion of the gonadotropins (Thibier and Rolland, 1976; Dubey and Plant, 1985; Rivier et al., 1986; Hayashi and Moberg, 1987; Juniewicz et al., 1987; Saketos et al., 1993; Turner et al., 1999b). In contrast, central administration of a synthetic salt, 1-adamantanecarbonyl-Arg-Phe-NH2 trifluoroacetate (RF9) stimulates gonadotropins in rodents as well as sheep (Pineda et al., 2010; Caraty et al., 2012; Rizwan et al., 2012). Furthermore, our previous study found that fasting-induced stress repressed HPG-axis in monkeys while RF9 administration rescued fasting-induced repressed HPG-axis (Batool et al., 2014). Originally, RF9 has been used as a neuropeptide FF receptors (NPFFRs) (NPFF1R or GPR147 and NPFF2R or GPR74) antagonist (Simonin et al., 2006). This compound potently blocked NPFFRs-mediated increase in heart rate and blood pressure and prevented opioid-induced hyperalgesia and tolerance in rats (Simonin et al., 2006). Gonadotropin inhibitory hormone (GnIH) inhibits HPG-axis via GPR147 signaling, therefore, initially RF9 has been used as an antagonist of GnIH to activate HPG-axis (Pineda et al., 2010; Caraty et al., 2012; Rizwan et al., 2012; Ullah et al., 2016). However, few recent studies found that independent of GnIH, RF9 directly binds with GPR54 (kisspeptin receptor) and stimulates gonadotropin release (Liu and Herbison, 2014; Min et al., 2015). These studies reported that RF9 failed to activate GnRH neurons and gonadotropin secretions in GPR54 knock-out set up (Liu and Herbison, 2014; Min et al., 2015).

Cortisol inhibits HPG-axis (Thibier and Rolland, 1976; Dubey and Plant, 1985; Rivier et al., 1986; Hayashi and Moberg, 1987; Juniewicz et al., 1987; Saketos et al., 1993; Turner et al., 1999b), whereas; RF9 administration activates HPG-axis (Batool et al., 2014). These data suggest that RF9 may rescue cortisol-mediated repressed HPG-axis. To prove our hypothesis, we injected hydrocortisone to adult male rhesus monkeys and found repression of HPG-axis. To investigate whether RF9 rescues cortisol-induced repressed HPG-axis, we injected RF9 to hydrocortisone treated monkeys and rescued cortisol-induced repressed HPG-axis. Our results suggest that RF9 rescues cortisol-induced repression of the HPG-axis.

## Materials and Methods

### Animals

Six adult intact male rhesus monkeys (*Macaca mulatta*), weighing 10.10 ±0.46 kg, were used in this study. The experiment was approved by the Departmental Committee for Care and Use of Animals, Department of Animal Sciences, Quaid-i-Azam University, Islamabad, Pakistan. Information on feeding, housing, sedation, and chair-restraint-training of the experimental animals has been discussed in our previously published studies (Wahab et al., 2008, 2010; Batool et al., 2014; Ullah et al., 2017).

### Venous Catheterization

To obtain sequential blood samples and administer RF9/vehicle, a Farcocath cannula (0.9 mm/22G; Medical Industries SAE Alexandria, Egypt) was placed in the saphenous vein of the animals under ketamine (5 mg/kg BW, im)-induced anesthesia as reported previously (Wahab et al., 2008, 2010; Batool et al., 2014; Ullah et al., 2017). This cannula was lined to the syringe through a butterfly tube (length 300 mm, volume 0.29 ml 20GX3/4″, JMS, Singapore). The experiment was not initiated until the animals had fully recovered from the sedation.

### Treatment

Animals were prepared as a stress model by administering hydrocortisone sodium succinate (Solucortef; Pfizer, Puurs, Belgium) diluted in normal saline (0.9% NaCl). Hydrocortisone was injected at the rate of 20mg/kg BW/day to each animal for 12 days. The total daily dose of hydrocortisone was given split equally into three im injections (at 9 am, 3 pm, and 9 pm). The dose of hydrocortisone was determined based upon previous experiments in rhesus monkeys, where the animals were treated for 62 days with hydrocortisone. The initial dose was 10 mg hydrocortisone/(kg BW/day) but this was subsequently increased after 7 days to 20 mg/(kg BW/day) (Dubey and Plant, 1985). As our treatment period was 12 days, therefore we selected 20 mg/(kg BW/day). On experimental day 12, a bolus of RF9 (0.1 mg/Kg BW) dissolved in normal saline containing 0.0012% DMSO (China peptides, Shanghai, China) was administered intravenously to three animals whereas normal saline containing 0.0012% DMSO was given to three animals as a vehicle. The dose of RF9 was based on our previous studies (Batool et al., 2014; Ullah et al., 2017).

### Blood Sampling

Single blood sample (1.5 ml) was obtained on alternate days during the 12 days hydrocortisone treatment from each animal. On experimental day 12, the animals were administered RF9 (*n* = 3) and vehicle (*n* = 3) at 1200 h and subjected to a 4 h sequential blood sampling at 60 min intervals from 12:00 to 16:00 h. The first sample was taken before RF9/vehicle administration. To maintain blood volume, following the withdrawal of each sample, an equal amount of heparinized (5 IU/ml) normal saline was infused. Collected samples were stored in ice-filled container till 17 h and were then centrifuged at 3500 rpm at 4°C for 15 min. Blood plasma was separated and stored at −20°C until hormones analyses.

### Pharmacological Agents

Hydrocortisone sodium succinate (Solucortef; Pfizer, Puurs, Belgium), ketamine, and heparin (Rotexmedica, Trittau, Germany) and normal saline (0.9% NaCl) were purchased locally. RF9 (1-Adamantanecarbonyl-Arg-Phe-NH2), a trifluoroacetate salt was synthesized by China peptides (Shanghai, China). Working solutions of RF9 were prepared in normal saline (0.9% NaCl) containing 0.0012% DMSO.

### Hormones Analysis

Cortisol and testosterone EIA kits (MicroLISA, Amgenix Inc., San Jose, CA, United States) were used to measure plasma testosterone and cortisol levels. Assays were carried out according to the manufacturer’s protocol. The intra and inter-assay variation coefficients of testosterone assay were lower than 10% and sensitivity of the assay was 0.05 ng/ml. The intra- and inter-assay variations coefficients for cortisol assay were <16% while its sensitivity was 1.5 ng/ml.

### Statistics

Mean plasma testosterone and cortisol concentrations before and after hydrocortisone treatment were assessed by performing paired *t*-test and one-way ANOVA with repeated measures. Variation in the mean testosterone concentration in response to saline and RF9 administration during stressed condition was measured by using two-way ANOVA. All data were expressed as mean ± SEM and *P* < 0.05 was taken as an indication of significant difference. Data were analyzed by using Graph Pad Prism version 5.01 (GraphPad Software Inc., San Diego, CA, United States). The raw data with the calculation of % change is shown in Supplementary Table 1. As there were big variations among animals within group and between groups, therefore % change has been used instead of original values.

## Results

### Hydrocortisone Treatment Increases Plasma Cortisol Levels

Cortisol is a terminal end product of the HPA-axis. To investigate that hydrocortisone treatment affect circulating cortisol, plasma cortisol levels were checked. All animals showed low plasma cortisol levels on day 1, which progressively increased with the hydrocortisone treatment. One-way ANOVA indicated that plasma cortisol levels were raised acutely (*P* ≤ 0.0001) on all post-treatment days compared to pre-treatment concentrations (Supplementary Figure 1A). Mean plasma cortisol levels after the treatment were significantly increased compared to the pre-treatment (day 1), as analyzed by paired *t*-test (Supplementary Figure 1B).

### Hydrocortisone Treatment Decreases Plasma Testosterone Levels

**FIGURE 1:**
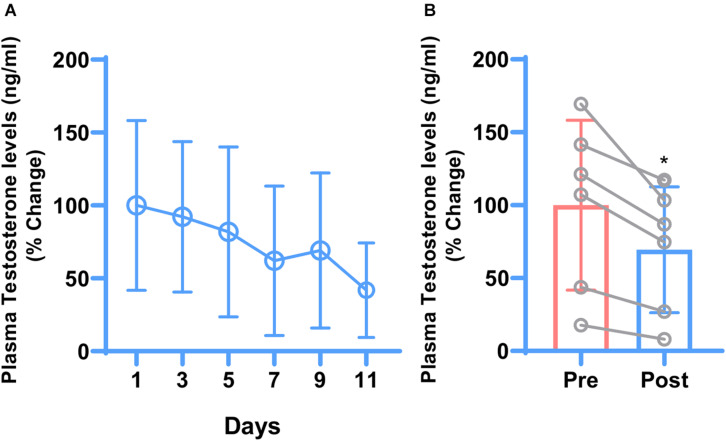
Daily changes in mean plasma testosterone concentrations during 11 days of hydrocortisone treatment in adult male rhesus macaque (*n* = 6). One-way ANOVA showed decreasing trend of testosterone levels but because of the big variations, the difference was not significant **(A)**. Similarly, paired *t*-test showed that hydrocortisone significantly (*P ≤ 0.0127) decreased the overall mean plasma testosterone **(B)**.

Testosterone is the terminal end product of the HPG-axis. To check whether hydrocortisone treatment affects the HPG-axis, plasma testosterone levels were checked. Opposite to cortisol, one-way ANOVA indicated that plasma testosterone levels showed a declining trend with the treatment (Figure 1A). Post-treatment mean plasma testosterone levels were significantly lowered (*P* ≤ 0.0127) compared to pre-treatment testosterone levels as indicated by paired *t*-test (Figure 1B).

### RF9 Administration Rescues Hydrocortisone-Induced Repression of the HPG-Axis

**FIGURE 2:**
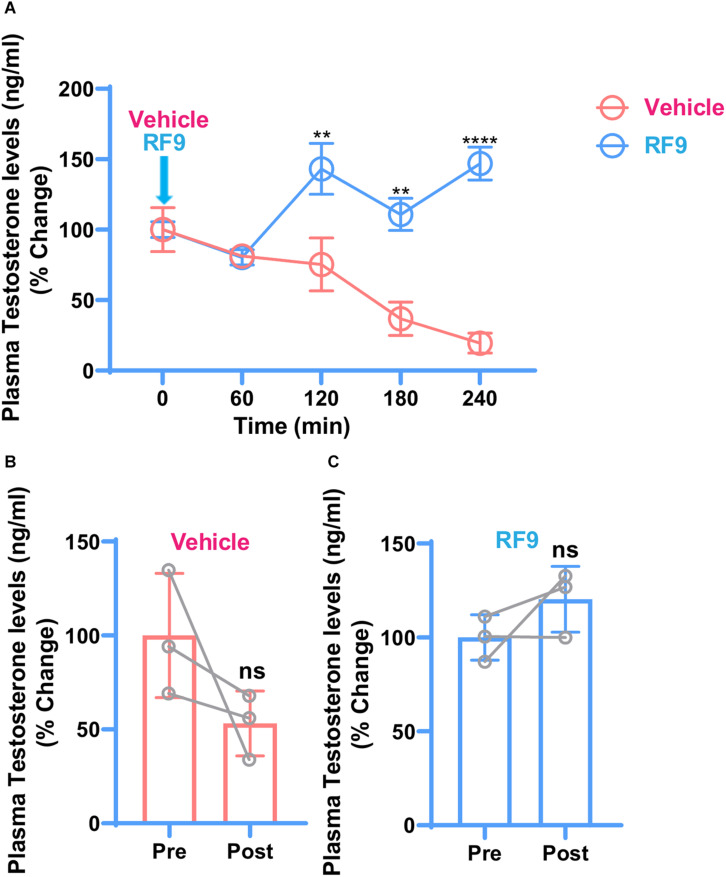
Changes in mean ± SEM plasma testosterone concentration as reflected by % change with respect to 0-min concentration, before and after iv administration of RF9/vehicle (arrow) in hydrocortisone treated adult male rhesus monkeys (*n* = 3). Two-way ANOVA with *post hoc* Bonferroni test showed a significant effect of treatment at 120 min (**P ≤ 0.0037), 180 min (**P ≤ 0.0016), and 240 min (****P ≤ 0.0001) intervals **(A)**. Decreasing and increasing trend in post-treatment plasma testosterone levels were observed in vehicle **(B)** and RF9 **(C)** groups, respectively. It seems that because of the variations within groups, there is no significant difference in post-treatment testosterone levels in **(B,C)**.

To investigate whether RF9 rescues cortisol-induced repression of the HPG-axis, RF9 was injected and testosterone levels were checked as an end product of the HPG-axis. Compared to vehicle, increased plasma testosterone levels were found in the RF9 injected animals. Two-way ANOVA revealed significantly increased plasma testosterone levels at 120 min (*P* ≤ 0.0037), 180 min (*P* ≤ 0.0016), and 240 min (*P* ≤ 0.0001) intervals in hydrocortisone treated monkeys. Our results are suggesting an acute stimulation of plasma testosterone by RF9 compared to vehicle. Mean plasma testosterone after RF9 and vehicle administration in the hydrocortisone treated monkeys are shown in Figure 2.

## Discussion

In the current study, the role of RF9 in activating cortisol-induced repressed HPG-axis in higher primates was investigated. A 12 days treatment with synthetic glucocorticoid, hydrocortisone dramatically increased plasma cortisol levels and suppressed the HPG-axis in the adult intact male rhesus monkeys, as evident by the inhibition of mean plasma testosterone levels. The repressed HPG-axis and activated HPA-axis have been reported in various animal models of stress (Rivier et al., 1986; Rivier and Rivest, 1991; Samuels and McDaniel, 1997; Wahab et al., 2008). Cortisol and testosterone are the terminal end products of the HPA-axis and the HPG-Axis, respectively. Various studies have shown that administration of natural or synthetic glucocorticoids inhibits the secretion of the gonadotropins in rats (Rivier et al., 1986), sheep (Juniewicz et al., 1987), pigs (Turner et al.,1999a,b), cattle (Thibier and Rolland, 1976), rhesus monkeys (Dubey and Plant, 1985; Hayashi and Moberg, 1987), and humans (Saketos et al., 1993). Our previous study found that fasting-induced stress inhibits HPG-axis whereas RF9 administration rescues it (Batool et al., 2014).

Based on the above evidences, we hypothesized that RF9 may rescue cortisol-induced repression of the HPG-axis. To answer the above question, we inhibited the HPG-axis by the administration of hydrocortisone and rescued the repressed HPG-axis by RF9 administration. The observed effects on plasma cortisol and testosterone were similar to the previous findings noted with this treatment in the rhesus macaque (Dubey and Plant, 1985). The most important result of the current study is that RF9 administration significantly relieved the suppressed plasma testosterone levels in the hydrocortisone treated monkeys. Originally, RF9 was used as a NPFF1R or GPR147 and NPFF2R or GPR74 antagonist that potently blocked the NPFF-induced increase in the blood pressure and heart rate (Simonin et al., 2006). GnIH signals through GPR147 and inhibits the HPG-axis (Ullah et al., 2016). Cortisol has receptors on kisspeptin, GnIH and GnRH neurons. Therefore, it is possible that cortisol regulates GnRH neuron directly or indirectly through kisspeptin and GnIH neurons. Recently, few studies reported that RF9 acts as a receptor (GPR54) agonist for kisspeptin (Liu and Herbison, 2014; Min et al., 2015). Liu and Herbison (2014) found that independent of GnIH, RF9 increases GnRH firing rate exclusively through GPR54 as GPR54 deletion from GnRH neurons failed to increase the firing frequency of GnRH neurons. Likewise, Min et al. (2015) found that RF9 failed to increase plasma LH levels in GPR54 −/− and GPR54/GPR147 −/− mice but it increased plasma LH levels in GPR147 −/− mice. The above data suggest that RF9 acts as an agonist of GPR54 as well as an antagonist of GPR74 and GPR147 but RF9-GPR54 interaction regulates HPG-axis whereas RF9-NPFFRs interaction regulates other functions including blood pressure and heart rate, however, it needs further studies. Furthermore, our results also suggest that cortisol represses HPG-axis predominantly by inhibiting kisspeptin and RF9 directly bind with GPR54 and activate HPG-axis. Being agonist of GPR54, our results suggest a possible contribution of the central endogenous kisspeptin signaling in mediating stress-induced suppression of the HPG axis in the adult male primates. Cortisol and kisspeptin affect HPG-axis at hypothalamic, pituitary and gonadal levels (Wahab et al., 2015; Ullah et al., 2016). Systemic administration of RF9 rescues cortisol-induced repression of the HPG-axis. We do not know which component of the HPG-axis is affected by cortisol and RF9; therefore, further studies are warranted to investigate the exact mode of action of cortisol and RF9 along the HPG-axis.

## Conclusion

In summary, hydrocortisone reduces plasma testosterone and increases plasma cortisol levels. However, RF9 administration rescues hydrocortisone-induced reduced plasma testosterone levels in the adult male rhesus monkey. The failure to measure LH means that we did not rule out a non-central effect of RF9, therefore, the authors suggest further studies to explore it. However, as testosterone is the end product of HPG-axis and its concentration reflects the status of HPG-axis especially LH, therefore, we can say that cortisol represses whereas RF9 rescues cortisol-induced repressed HPG-axis.

## Data Availability Statement

The original contributions presented in the study are included in the article/Supplementary Material, further inquiries can be directed to the corresponding author/s.

## Ethics Statement

The animal study was reviewed and approved by the Departmental Committee for Care and Use of Animals, Department of Animal Sciences, Quaid-i-Azam University, Islamabad, Pakistan.

## Author Contributions

RU, MS, and JF designed the experiments. RU, RN, AB, TR, and MW performed the experiments. RU, GN, and RN analyzed the data and wrote the manuscript. MS, JF, and FW edited the manuscript. All authors contributed to the article and approved the submitted version.

## Conflict of Interest

The authors declare that the research was conducted in the absence of any commercial or financial relationships that could be construed as a potential conflict of interest.

## Supplementary Material

Supplementary Figure 1 Changes in mean plasma cortisol concentration during 11 day treatment of hydrocortisone in the intact adult male rhesus monkeys (*n* = 6). Total daily dose (20 mg/kg BW) was split into three im injections (at 9 am, 3 pm, and 9 pm). One-way ANOVA with *post hoc* Bonferroni test showed that hydrocortisone treatment acutely increased mean plasma cortisol concentration in 11 days treatment (^*⁣*⁣**^P ≤ 0.0001) **(A)**. Similarly, paired *t*-test showed that hydrocortisone treatment has also significantly increased overall mean plasma cortisol concentration (^∗∗^*P* ≤ 0.0001) **(B)**, suggesting the stimulatory effect of hydrocortisone treatment on plasma cortisol.

Supplementary Table 1 Raw data of assays (Elisa) with the calculation of % change.
