# Supplementary figures and images for: RF9 Rescues Cortisol-Induced Repression of Testosterone Levels in Adult Male Macaques
Source: Front Physiol. 2021 Feb 25;12:630796. doi: 10.3389/fphys.2021.630796 (PMC7946976; doi:10.3389/fphys.2021.630796)

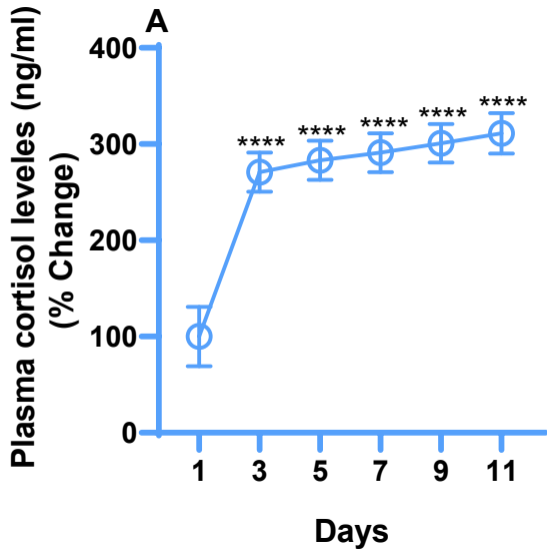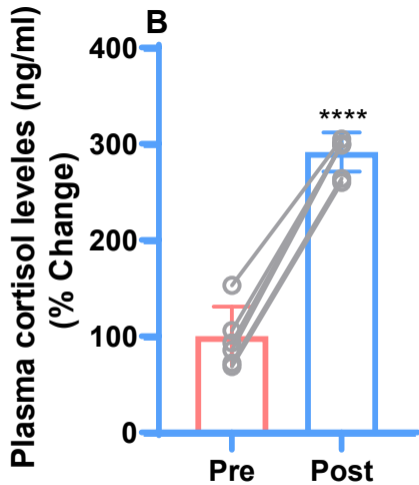

Supplement: Supplementary Figure 1 — Changes in mean plasma cortisol concentration during 11 day treatment of hydrocortisone in the intact adult male rhesus monkeys (n = 6). Total daily dose (20 mg/kg BW) was split into three im injections (at 9 am, 3 pm, and 9 pm). One-way ANOVA with post hoc Bonferroni test showed that hydrocortisone treatment acutely increased mean plasma cortisol concentration in 11 days treatment (*⁣*⁣**P ≤ 0.0001) (A). Similarly, paired t-test showed that hydrocortisone treatment has also significantly increased overall mean plasma cortisol concentration (∗∗P ≤ 0.0001) (B), suggesting the stimulatory effect of hydrocortisone treatment on plasma cortisol. [file Data_Sheet_1.PDF]
